# The correlation between the labrum size and the labral tear in asymptomatic volunteers and symptomatic patients

**DOI:** 10.1186/s13018-021-02719-5

**Published:** 2021-09-20

**Authors:** Guanying Gao, Qiang Fu, Ruiqi Wu, Rongge Liu, Yingfang Ao, Yan Xu

**Affiliations:** 1 Institute of Sports Medicine, Beijing Key Laboratory of Sports Injuries, Peking University Third Hospital, 49 North Garden Road, Haidian District, Beijing, 100191 China; 2 Department of Ultrasound, Peking University Third Hospital, 49 North Garden Road, Haidian District, Beijing, 100191 China

**Keywords:** Labral size, Labral tear, Hip, Magnetic resonance imaging

## Abstract

**Background:**

Some studies have proved that labrum size is associated with symptoms in patients with hip labral tear. The correlation between the labrum size and the labral tear in asymptomatic volunteers and symptomatic patients is still uncertain.

**Methods:**

The volunteers with no history of pain, injury, or surgery were recruited from the community. Patients who were diagnosed with labral tear and underwent hip arthroscopic surgery in this period in our hospital were also included. The length and height of the acetabular hip labrum were measured at three separate anatomic sites through magnetic resonance imaging (MRI) along the acetabular rim: lateral, anterior, and anteroinferior.

**Results:**

A total of 70 volunteers (125 hips) and 70 patients (70 hips) were included in this study. Sixty-six (52.8%) hips had labral tears in all 125 hips of volunteers. The lateral labral length of volunteers with labral tears was significantly larger than those without labral tears (*P* < .05). In 14 volunteers with unilateral labral tears, length of lateral, anterior, and anteroinferior labrum in the side with tear were significantly larger than normal on the other side. The anterior labral height of volunteers was significantly larger than that of patients (*P* < .05).

**Conclusions:**

In conclusion, asymptomatic volunteers with larger length of lateral, anterior, and anteroinferior labrum are more prone to present with labral tears. Symptomatic patients with labral tears exhibited thinner anterior labrum. Further studies are warranted to explore the mechanisms of labral tears in asymptomatic people and validate the use of labral size as a guide to differential diagnosis and treatment.

## Introduction

The acetabular labrum is a triangular fibrocartilaginous structure attached to the rim of the acetabulum and envelopes the femoral head, which creates a suction seal [1]. It may improve joint lubrication within the central compartment, increase the depth of the acetabulum, and increase joint stability [2, 3]. The size of labrum can be evaluated by magnetic resonance imaging (MRI) and intraoperative assessment. Previous study had proved a strong agreement between radiologic and arthroscopic measurement of labrum width when using MRI [4]. The current researches have proved a correlation existing between lateral acetabular coverage and labral length and labral size is significantly larger in dysplastic hips compared with nondysplastic hips [5, 6]. Tomoyuki et al. [7] proved that patients with symptomatic hips had significantly larger labrums than labrums in asymptomatic hips in the other side. However, there is no study comparing the size of the labrum between asymptomatic volunteers and symptomatic patients.

In our daily work, we noticed that patients with large labrum may be more prone to undergo labral tear. We hypothesized that labrum size may relate with labral tear and show difference in asymptomatic volunteers and symptomatic patients. The purpose of this study was to evaluate the correlation between the labrum size and the labral tear in asymptomatic volunteers and symptomatic patients.

## Methods

### Volunteers and patients

Volunteers with no symptoms were recruited from the community. The volunteers with no history of hip pain, injury, or surgery were included in this study. Volunteers chose bilateral MRI or random unilateral MRI of their own accord. Participants were excluded if they had claustrophobia or a contraindication to obtain an MRI. Patients who were diagnosed with labral tear and underwent hip arthroscopic surgery in this period in our hospital were also included. All patients had preoperative MRI of the affected side. All participants signed informed consent. The study was approved by the Ethics Committee of the Third Hospital of Peking University. All methods were performed in accordance with the guidelines and regulations of the Ethics Committee of the Third Hospital of Peking University.

#### MRI

The hip magnetic resonance examinations were performed as previously described [8], with a 3.0T magnetic resonance scanner (Magnetom Trio with TIM system, Siemens Healthcare, Erlangen, Germany) and a dedicated flexible surface coil around the affected hip joint. The patients were in the supine position. The MRI hip protocol consisted of an axial fat-saturated proton density (FSPD) sequence, coronal FSPD sequences, and an oblique sagittal FSPD sequence. Imaging in the oblique sagittal plane was performed parallel to the axis of the femoral neck.

#### Measurements

**Fig. 1:**
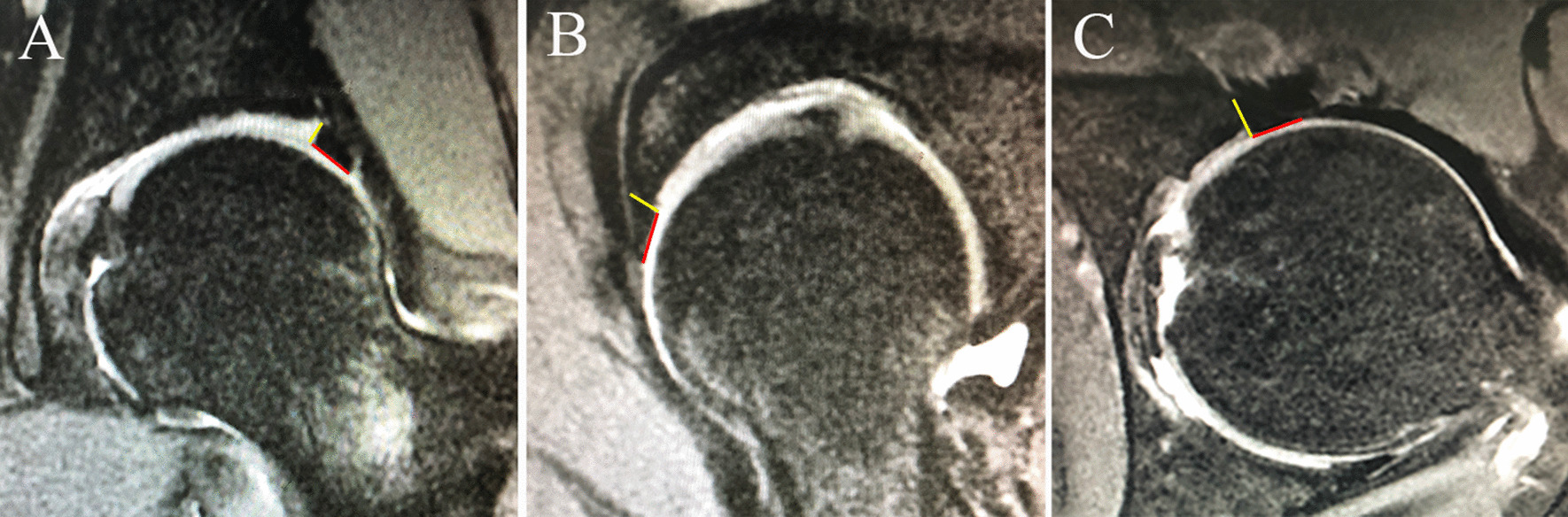
The labral length was measured from the chondrolabral junction to the tip of the labrum and labral height was the distance of the attachment measured from the joint surface to the perilabral recess utilizing a fat-saturated proton density (FSPD) sequence. The red line presents the length of the labrum and the yellow line presents the height of labrum. **A** Lateral labral length and height measurement technique on coronal magnetic resonance imaging (MRI). **B** Anterior labral length and height measurement technique on oblique sagittal MRI. **C** Anteroinferior labral length and height measurement technique on axial MRI

The measurements of labral size were made independently by two musculoskeletal radiologists, blinded to the clinical information and arthroscopic findings. The length and height of the acetabular hip labrum were measured at three separate anatomic sites along the acetabular rim: lateral, anterior, and anteroinferior [6, 9]. The labral length was measured from the chondrolabral junction to the tip of the labrum and labral height was the distance of the attachment measured from the joint surface to the perilabral recess on a picture archiving and communication systems (PACS) workstation (Fig. 1). Alpha angle and lateral center–edge angle (LCEA) were calculated as described by previous studies [10, 11].

#### Statistics

Intra- and interclass correlation coefficients were calculated to examine the reproducibility of measurements of labral size. MRI measurement of labral size at three separate anatomic sites was performed twice by one radiologist and once by another radiologist. The interclass correlation coefficient was calculated from the mean of the first two measurements performed by the initial investigator and the single measurement of the verifying investigator. The two-tailed paired t test was used to evaluate significance between labral size of hips in patients with unilateral tear. Continuous variables with a normal distribution in the baseline data between groups were examined using the independent-samples t test. Percentages were compared using the Chi-square test. *P* values < 0.05 were considered statistically significant. All statistical analyses were performed with SPSS Statistics, version 22 (IBM).

## Results

As shown in Table 1, a total of 70 volunteers (125 hips) and 70 patients (70 hips) were included in this study. Fifty-five volunteers underwent bilateral MRI and 15 volunteers underwent unilateral MRI. The mean age of the asymptomatic volunteers was 35.6 years (range, 26–48 years), and the mean age of patients was 33.1 years (range, 20–44 years). The mean body mass index (BMI) of volunteers was 23.5 (range, 19.0–30.1), and mean BMI of patients was 23.4 (range, 18.8–29.2). The age and BMI difference between the groups was not statistically significant (*P* > 0.05). There was no significant difference between BMI and labral size both in volunteers group and patients group. There were 36 (51.4%) females and 34 (48.6%) males in the asymptomatic volunteer group, and 30 (42.9%) females and 40 (57.1%) males in the patient group. Sixty-six (52.8%) hips had labral tears in all 125 hips of volunteers. Fourteen volunteers had unilateral tears. Fifty-two patients (74.3%) of the 70 patients had pincer femoroacetabular impingement (FAI), and 61 patients (87.1%) had cam-type FAI and all patients had labral tears. The mean alpha angle of asymptomatic volunteers and symptomatic patients were 49.5 ± 7.1 (range, 38.9–66.0) and 61.5 ± 4.9 (range, 55.1–70.6), respectively. The mean LCEA of asymptomatic volunteers and symptomatic patients were 28.0 ± 5.7 (range, 20.0–39.7) and 30.9 ± 3.3 (range, 27.3–35.7), respectively. Asymptomatic volunteers with borderline dysplasia (LCEA 20°–24.9°) had larger lateral labral length compared with asymptomatic volunteers with normal acetabular coverage (LCEA 25°–39.9°) (*P* < 0.05). There was no significant correlation between alpha angle and labral size.

**Table 1:** Demography of VOLUNTEERS (*n* = 70) and patients (*n* = 70)

Parameter	Data
*Volunteers*	
Age, y (range)	35.6 (26–48)
BMI, kg/m^2^ (range)	23.5 (19.0–30.1)
Male	34 (48.6%)
Female	36 (51.4%)
Side	
Left	55 (44%)
Right	70 (56%)
*Alpha angle (range)*	49.5 (38.9–66.0)
*LCEA (range)*	28.0 (20.0–39.7)
Labral tear, hips	66 (52.8%)
*Patients*	
Age, y (range)	33.1 (20–44)
BMI, kg/m^2^ (range)	23.4 (18.8–29.2)
Male	40 (57.1%)
Female	30 (42.9%)
Side	
Left	31 (44.3%)
Right	39 (55.7%)
*Alpha angle (range)*	61.5 (55.1–70.6)
*LCEA (range)*	30.9 (27.3–35.7)
Labral tear	70 (100%)
Cam impingement	61 (87.1%)
Pincer impingement	52 (74.3%)

Unless otherwise specified, data are numbers of patients, with percentages in parentheses

As shown in Table 2, the mean lateral labral length, lateral labral height, anterior labral length, anterior labral height, anteroinferior labral length, and anteroinferior labral height of hips in volunteers were 5.90 ± 1.69, 4.43 ± 1.05, 7.99 ± 1.73, 4.26 ± 0.82, 5.95 ± 2.02, 4.20 ± 0.90, respectively. In patients, the mean lateral labral length, lateral labral height, anterior labral length, anterior labral height, anteroinferior labral length and anteroinferior labral height of hips without labral tear were 5.64 ± 2.19, 4.75 ± 1.33, 7.66 ± 2.20, 3.55 ± 0.79, 5.87 ± 1.29, 4.73 ± 0.97, respectively. There was significant difference in anterior labral height between volunteers and patients (*P* < 0.05).

**Table 2:** Labral size of volunteers and patients

Groups	Labral size					
	Lateral		Anterior		Anteroinferior	
	Length	Height	Length	Height	Length	Height
Volunteers	5.90 ± 1.69	4.43 ± 1.05	7.99 ± 1.73	4.26 ± 0.82^α^	5.95 ± 2.02	4.20 ± 0.90
Patients	5.64 ± 2.19	4.75 ± 1.33	7.66 ± 2.20	3.55 ± 0.79^α^	5.87 ± 1.29	4.73 ± 0.97
Labrum in volunteers with tear	6.46 ± 1.79^β^	4.41 ± 1.04	8.07 ± 1.86	4.40 ± 0.76	5.75 ± 2.11	4.10 ± 0.94
Labrum in volunteers without tear	5.44 ± 1.47^β^	4.45 ± 1.06	7.92 ± 1.64	4.14 ± 0.87	6.10 ± 1.92	4.27 ± 0.88
Labrum in the side with tear in volunteers with unilateral tear	5.86 ± 1.27^γ^	4.36 ± 1.04	7.57 ± 2.01^δ^	3.77 ± 0.62	5.88 ± 1.47^ε^	4.55 ± 0.70
Labrum in the side without tear in volunteers with unilateral tear	5.00 ± 1.18^γ^	4.23 ± 0.98	6.91 ± 1.46 ^δ^	3.85 ± 0.92	6.63 ± 1.38 ^ε^	4.39 ± 0.71

Values are the mean ± SD. *α*, *β*, *γ*, *δ* and *ε* show significant difference between two values

The mean lateral labral length, lateral labral height, anterior labral length, anterior labral height, anteroinferior labral length and anteroinferior labral height of hips with labral tear in volunteers were 6.46 ± 1.79, 4.41 ± 1.04, 8.07 ± 1.86, 4.40 ± 0.76, 5.75 ± 2.11, 4.10 ± 0.94, respectively. The mean lateral labral length, lateral labral height, anterior labral length, anterior labral height, anteroinferior labral length and anteroinferior labral height of hips without labral tear in volunteers were 5.44 ± 1.47, 4.45 ± 1.06, 7.92 ± 1.64, 4.14 ± 0.87, 6.10 ± 1.92, 4.27 ± 0.88, respectively. There was significant difference in lateral labral length between volunteers with and without labral tear (*P* < 0.05).

There were 14 volunteers who had unilateral labral tears. In volunteers with unilateral labral tears, the mean lateral labral length, lateral labral height, anterior labral length, anterior labral height, anteroinferior labral length and anteroinferior labral height of labrum in the side with tear were 5.86 ± 1.27, 4.36 ± 1.04, 7.57 ± 2.01, 3.77 ± 0.62, 5.88 ± 1.47, 4.55 ± 0.70, respectively. The mean lateral labral length, lateral labral height, anterior labral length, anterior labral height, anteroinferior labral length and anteroinferior labral height of labrum in the side without tear were 5.00 ± 1.18, 4.23 ± 0.98, 6.91 ± 1.46, 3.85 ± 0.92, 6.63 ± 1.38, 4.39 ± 0.71, respectively. There was a significant difference in length of lateral, anterior and anteroinferior labrum between labrum in the side with and without tear in volunteers with unilateral labral tears (*P* < 0.05). The intraclass correlation coefficients between the two measurements made by the same observer were 0.96, 0.86, 0.81, 0.84, 0.90, and 0.86 for measurements of labrum length at lateral, anterior, and anteroinferior hip and height at these three separate anatomic sites, respectively. The interclass correlation coefficients were 0.92, 0.79, 0.79, 0.76, 0.81, and 0.81 for measurements of labrum length at lateral, anterior, and anteroinferior hip and height at these 3 separate anatomic sites, respectively.

## Discussion

In this study, we found that larger labrum was more likely to tear in asymptomatic volunteers. Lateral labral length of hips with labral tear of volunteers were significantly larger than those without labral tear. Length of lateral, anterior and anteroinferior labrum in the side with tear in volunteers with unilateral labral tears were significantly larger than those without tear in the other side. However, there was no significant difference in labral length between asymptomatic volunteers and symptomatic patients. It is just that anterior labral height of volunteers was significantly larger than those of patients. Sixty-six (52.8%) hips had labral tears in all 125 hips of volunteers.

Over the past decade, hip arthroscopic surgery has developed rapidly and has been proved to have good clinical outcomes in the treatment of FAI and combined labral tears in both adults and adolescents [12–15]. Hip chondral lesions associated with FAI could be also treated by microfracture, autologous matrix-induced chondrogenesis and matrix-induced autologous chondrocyte implantation [16–18]. It was also well studied that frankly dysplastic and borderline dysplastic hips exhibited larger values of labral length at all locations when compared with hips with normal acetabular coverage or acetabular overcoverage [5–7, 19]. Increased shear stress to the acetabular margin may evolve into hypertrophy or tear of the labrum [20]. In this study, asymptomatic volunteers with borderline dysplasia had larger lateral labral length compared with asymptomatic volunteers with normal acetabular coverage (*P* < 0.05), which was consistent with previous studies. Garabekyan et al. [6] evaluated 236 patients and concluded that patients with borderline dysplasia and frank dysplasia exhibited increased values of labral length and the size of the pincer or cam lesion, degree of femoral torsion, degree of acetabular version, and BMI did not bear significant correlations to labral length. Tomoyuki et al. [7] evaluated 102 patients and concluded that acetabular labral length is significantly larger in dysplastic, irregularly congruent and symptomatic hips. However, that study did not conduct a comparison between symptomatic patients and people with no symptoms on both hips and valuated only the length of the labrum without considering its volume or thickness. Besides, that study only evaluated the lateral labral length of the coronal plane. We though evaluation in the sagittal and axial plane can help better understand the effect of labrum morphology. In our study, we used asymptomatic volunteers as control group and the length and thickness of the labrum at three locations were all evaluated.

There are several studies on the topic of asymptomatic labral tears, but with quite different findings. Lee et al. [21] performed MRI in 70 asymptomatic volunteers aged 19–41 years and found labral tears in 39%. Register et al. [22] evaluated 45 asymptomatic volunteers and revealed abnormalities in 73% of hips using MRI, with labral tears being identified in 69% of the joints. Tresch et al. [23] evaluated 63 asymptomatic volunteers using MRI and labral tears were detected in 36.5% for reader 1 and 52.4% for reader 2. Silvis et al. [24] evaluated 21 professional and 18 collegiate asymptomatic hockey players and found that acetabular labral tear was reported in 22 of 39 athletes (56%). Aydingöz et al. [25] evaluated 360 hips in 180 volunteers and found percentage of volunteers with intralabral intensity increases on T2-weighted gradient-echo MR images by age (from 10.7 to 63.3%). Schmitz et al. [26] found labral tears on MR imaging in 81–86% of 42 asymptomatic hips. Gallo et al. [27] evaluated 21 professional hockey players with no previous hip/groin pain and found 15 (71.4%) had labral tears identified in 1 or both hips. Nineteen of 21 players (90%) in that study continued to play professional hockey at 4 years' follow-up. The development of any hip and/or pelvis symptoms occurred in only 3 players (14%) within 4 years. The authors thought hip pathology is commonly uncovered on MRI of asymptomatic hockey players. However, this pathology does not produce symptoms or result in missed games within 4 years in most players. We thought it might be the same in asymptomatic volunteers. Although there is a high prevalence of labral tear in asymptomatic volunteers and players, this pathology may not produce symptoms. In our study, 66 (52.8%) hips of all the 125 hips had labral tear, which is moderate compared with the studies mentioned above. In consideration of high percentage of asymptomatic MRI findings of labral tears, we need to pay attention to whether the patient's labral tear is the cause of symptoms, so as to avoid missed diagnosis of other causes of symptoms. Through further study, the size of the labrum may be an indicator of differential diagnosis.

Until recently there was no mention of the mechanism of labral tear in nondysplastic hips and hips without FAI [28, 29]. In our study, the longer and thinner labrum seem to tear more easily. The longer labrum has a larger area and may be more likely to be damaged. The acetabular labrum provides stability to distraction forces through the suction effect of the hip fluid seal and labral tear can damage suction effect [30]. We thought suction effect may be damaged more easily in patients with thinner labrum, which resulted in symptoms. Another reason why patients who underwent surgery had thinner labrum than volunteers may be that inflammation and damage after injury make labrum thinner. Further research is needed to identify the mechanism of labral tear in volunteers and the influence of labral size on labral tear and symptoms.

### Limitations

This study has several limitations. Firstly, we did not have data about intraoperative measurement of the labral length in patients group. Correlation between more accurate intraoperative measurement of labral length and our MRI measurement could not be evaluated. Secondly, this study did not evaluate interobserver reproducibility of MRI measurements. Thirdly, this study did not quantify the relationship between symptoms and the size of the labrum in patients.

## Conclusions

Asymptomatic volunteers with larger length of lateral, anterior and anteroinferior labrum are more prone to present with labral tears. Symptomatic patients with labral tears exhibited thinner anterior labrum. Further studies are warranted to explore the mechanisms of labral tears in asymptomatic people and validate the use of labral size as a guide to differential diagnosis and treatment.

## Data Availability

Not applicable.

## Abbreviation

FAI: femoroacetabular impingement

## References

[1] Seldes RM, Tan V, Hunt J, Katz M, Winiarsky R, Fitzgerald RH (2001). Anatomy, histologic features, and vascularity of the adult acetabular labrum. Clin Orthop Relat Res.

[2] Bowman KF, Fox J, Sekiya JK (2010). A clinically relevant review of hip biomechanics. Arthroscopy.

[3] Crawford MJ, Dy CJ, Alexander JW, Thompson M, Schroder SJ, Vega CE (2007). The 2007 Frank Stinchfield Award. The biomechanics of the hip labrum and the stability of the hip. Clin Orthop Relat Res.

[4] Kaplan DJ, Samim M, Burke CJ, Meislin RJ, Youm T (2020). Validity of magnetic resonance imaging measurement of hip labral width compared with intraoperative assessment. Arthroscopy.

[5] Gupta A, Chandrasekaran S, Redmond JM, Hammarstedt JE, Cramer TL, Liu Y (2015). Does labral size correlate with degree of acetabular dysplasia?. Orthop J Sports Med.

[6] Garabekyan T, Ashwell Z, Chadayammuri V, Jesse MK, Pascual-Garrido C, Petersen B (2016). Lateral acetabular coverage predicts the size of the hip labrum. Am J Sports Med.

[7] Kamenaga T, Hayashi S, Hashimoto S, Takayama K, Niikura T, Kuroda R (2020). Larger acetabular labrum is associated with hip dysplasia, joint incongruency and clinical symptom. Arthroscopy.

[8] Gao G, Fu Q, Cui L, Xu Y (2019). The diagnostic value of ultrasound in anterosuperior acetabular labral tear. Arthroscopy.

[9] Toft F, Anliker E, Beck M (2015). Is labral hypotrophy correlated with increased acetabular depth?. J Hip Preserv Surg.

[10] Stelzeneder D, Hingsammer A, Bixby SD, Kim YJ (2013). Can radiographic morphometric parameters for the hip be assessed on MRI?. Clin Orthop Relat Res.

[11] Hack K, Di Primio G, Rakhra K, Beaule PE (2010). Prevalence of cam-type femoroacetabular impingement morphology in asymptomatic volunteers. J Bone Jt Surg Am.

[12] Migliorini F, Maffulli N, Knobe M, Eschweiler J, Tingart M, Baroncini A. Arthroscopic labral repair for femoroacetabular impingement: a systematic review. Surgeon. 2021. 10.1016/j.surge.2021.02.013. 10.1016/j.surge.2021.02.013 33820729

[13] Migliorini F, Maffulli N. Arthroscopic management of femoroacetabular impingement in adolescents: a systematic review. Am J Sports Med. 2021. 10.1177/0363546521997138. 10.1177/0363546521997138 33740385

[14] Migliorini F, Liu Y, Eschweiler J, Baroncini A, Tingart M, Maffulli N. Increased range of motion but otherwise similar clinical outcome of arthroscopy over open osteoplasty for femoroacetabular impingement at midterm follow-up: a systematic review. Surgeon. 2021. 10.1016/j.surge.2021.01.016. 10.1016/j.surge.2021.01.016 33731304

[15] Fioruzzi A, Acerbi A, Jannelli E, Ivone A, Fontana A (2020). Interobserver and intraobserver reliability of a new radiological classification for femoroacetabular impingement syndrome. Musculoskelet Surg.

[16] de Girolamo L, Jannelli E, Fioruzzi A, Fontana A (2018). Acetabular chondral lesions associated with femoroacetabular impingement treated by autologous matrix-induced chondrogenesis or microfracture: a comparative study at 8-year follow-up. Arthroscopy.

[17] Jannelli E, Fontana A (2017). Arthroscopic treatment of chondral defects in the hip: AMIC, MACI, microfragmented adipose tissue transplantation (MATT) and other options. SICOT J.

[18] Jannelli E, Parafioriti A, Acerbi A, Ivone A, Fioruzzi A, Fontana A (2019). Acetabular delamination: epidemiology, histological features, and treatment. Cartilage.

[19] Petersen BD, Wolf B, Lambert JR, Clayton CW, Glueck DH, Jesse MK (2016). Lateral acetabular labral length is inversely related to acetabular coverage as measured by lateral center edge angle of Wiberg. J Hip Preserv Surg.

[20] Vendittoli PA, Young DA, Stitson DJ, Wolfe R, Del Buono A, Maffulli N (2012). Acetabular rim lesions: arthroscopic assessment and clinical relevance. Int Orthop.

[21] Lee AJ, Armour P, Thind D, Coates MH, Kang AC (2015). The prevalence of acetabular labral tears and associated pathology in a young asymptomatic population. Bone Jt J.

[22] Register B, Pennock AT, Ho CP, Strickland CD, Lawand A, Philippon MJ (2012). Prevalence of abnormal hip findings in asymptomatic participants: a prospective, blinded study. Am J Sports Med.

[23] Tresch F, Dietrich TJ, Pfirrmann CWA, Sutter R (2017). Hip MRI: Prevalence of articular cartilage defects and labral tears in asymptomatic volunteers. A comparison with a matched population of patients with femoroacetabular impingement. J Magn Reson Imaging.

[24] Silvis ML, Mosher TJ, Smetana BS, Chinchilli VM, Flemming DJ, Walker EA (2011). High prevalence of pelvic and hip magnetic resonance imaging findings in asymptomatic collegiate and professional hockey players. Am J Sports Med.

[25] Aydingoz U, Ozturk MH (2001). MR imaging of the acetabular labrum: a comparative study of both hips in 180 asymptomatic volunteers. Eur Radiol.

[26] Schmitz MR, Campbell SE, Fajardo RS, Kadrmas WR (2012). Identification of acetabular labral pathological changes in asymptomatic volunteers using optimized, noncontrast 1.5-T magnetic resonance imaging. Am J Sports Med.

[27] Gallo RA, Silvis ML, Smetana B, Stuck D, Lynch SA, Mosher TJ (2014). Asymptomatic hip/groin pathology identified on magnetic resonance imaging of professional hockey players: outcomes and playing status at 4 years' follow-up. Arthroscopy.

[28] Groh MM, Herrera J (2009). A comprehensive review of hip labral tears. Curr Rev Musculoskelet Med.

[29] Guevara CJ, Pietrobon R, Carothers JT, Olson SA, Vail TP (2006). Comprehensive morphologic evaluation of the hip in patients with symptomatic labral tear. Clin Orthop Relat Res.

[30] Nepple JJ, Philippon MJ, Campbell KJ, Dornan GJ, Jansson KS, LaPrade RF (2014). The hip fluid seal—part II: the effect of an acetabular labral tear, repair, resection, and reconstruction on hip stability to distraction. Knee Surg Sports Traumatol Arthrosc.

